# Incidentally Detected Thoracic Dumbbell Schwannoma With Intraspinal Extension and Cord Compression: A Case Report

**DOI:** 10.7759/cureus.110022

**Published:** 2026-06-01

**Authors:** T. A. M. S Wickramasinghe, Saman P Iddagoda, Dilip C Madanayake, Madhawa S Rathnaweera

**Affiliations:** 1 Surgery, Postgraduate Institute of Medicine, Colombo, LKA; 2 Thoracic Surgery, National Hospital for Respiratory Diseases, Welisara, LKA

**Keywords:** dumbbell tumour, neurology, neurosurgery, schwannoma, spinal surgery, thoracic, thoracic surgery

## Abstract

Schwannomas are benign neurogenic tumors commonly arising in the posterior mediastinum. Although typically asymptomatic, lesions with intraspinal extension may lead to neurological compromise. We report a 47-year-old male who presented with symptoms suggestive of a lower respiratory tract infection and was incidentally found to have a thoracic mass on chest radiography. Further imaging revealed a large, left paravertebral tumor with intrathoracic and intraspinal components extending through the left T7-T8 intervertebral foramina, consistent with a dumbbell schwannoma causing spinal cord compression. Options for surgery include thorax-first, spine-first, combined thoracic-neurosurgical open approaches, thoracoscopic-assisted approaches, and selected minimally invasive paraspinal or transforaminal techniques. Complete macroscopic excision was performed through a coordinated thoracic and spinal surgical approach involving open thoracotomy and foraminotomy. The patient's postoperative period was complicated by a lower respiratory tract infection requiring intravenous antibiotics. However, the patient was neurologically intact at post-op and at one-month follow-up. This case report highlights that large, thoracic dumbbell schwannomas may remain neurologically silent despite significant intraspinal extension and are best managed with a multidisciplinary approach to achieve the best outcomes.

## Introduction

Neurogenic tumors constitute the majority of posterior mediastinal masses, with schwannomas being the most common subtype [1,2]. These tumors arise from Schwann cells and are typically benign and slow-growing [2,3]. Thoracic schwannomas usually remain asymptomatic till they become large and hence can be discovered incidentally [1,3]. Large schwannomas usually present with cough, dyspnea, chest pain, and upper back pain due to increasing mass effect within the thorax [1,3]. Schwannomas extending through intervertebral foramina--“dumbbell tumors”--have an incidence of around 5% [4]. They can have neurological manifestations due to spinal cord compression [5,6]. In a targeted review of case reports, only a small number of thoracic dumbbell schwannoma cases could be clearly classified by preoperative neurological status, with both neurologically intact and neurologically symptomatic presentations being identified [6-11]. When thoracic schwannomas involve both intrathoracic and intraspinal components, their management often requires a multidisciplinary approach, with particular emphasis on timely intervention and meticulous operative planning in order to carry out excision while preserving the patient’s initial neurological status [9,12]. The objectives of the study are twofold: first, to highlight that this patient had an incidentally discovered massive schwannoma that had no obvious symptoms, even with a significant intraspinal component, and that a timely multidisciplinary approach allowed macroscopic excision while preserving his neurologically intact status.

## Case presentation

A 47-year-old male presented with symptoms suggestive of a lower respiratory tract infection. Chest radiography revealed a large opacity in the left hemithorax. Magnetic resonance imaging was done to further elucidate the characteristics of the lesion. It showed a posterior mediastinal mass with dimensions of 103 mm × 85 mm × 80 mm and an extension into the spinal canal. The mass extended through the D7/D8 foramen with a 32 mm × 22 mm × 22 mm extradural component in the spinal canal, causing effacement and compression of the spinal cord (Figure 1). Further questioning revealed a history of chronic back pain. However, history and examination did not reveal any motor, sensory, or autonomic manifestations of the tumor. A preoperative diagnosis of thoracic dumbbell schwannoma arising from the D8 nerve root was made.

**Figure 1:**
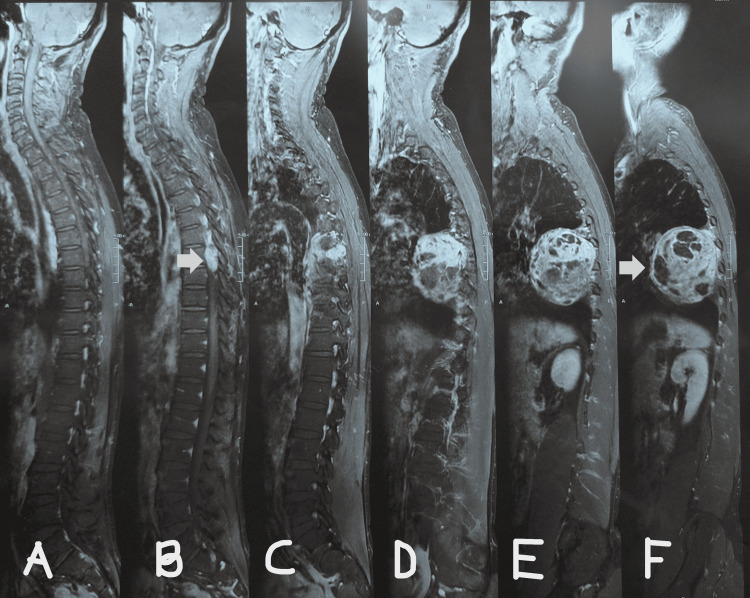
Magnetic resonance imaging - sagittal view showing the intraspinal (arrow) and intrathoracic (arrow) components of the dumbbell schwannoma Panel A - tumor not visualized on the right parasagittal section. Panel B - intraspinal component visualized on the left parasagittal section. Panel C - tumor emerging from the intervertebral foramen on the left parasagittal section. Panels D-F - intrathoracic portion of the tumor on the left parasagittal section.

The surgery was performed electively under general anaesthesia with single lung ventilation. A left posterolateral thoracotomy was performed. A large, intrathoracic, encapsulated mass was first excised macroscopically completely by the thoracic surgical team, and a level 9 lymph node was sampled for pathological analysis. This was done due to its proximity to the lesion and the need to exclude associated nodal pathology. There was no extension to the surrounding structures. Then, the intraspinal component of the excision was done by the spinal surgery team (Figure 2). Careful foraminotomy was done using up-biting ronguers and the entire macroscopic intraspinal component of the dumbbell tumor was dissected without breaching the dura (Figure 3). The intraspinal component was removed piecemeal to minimize traction on the spinal cord.

**Figure 2:**
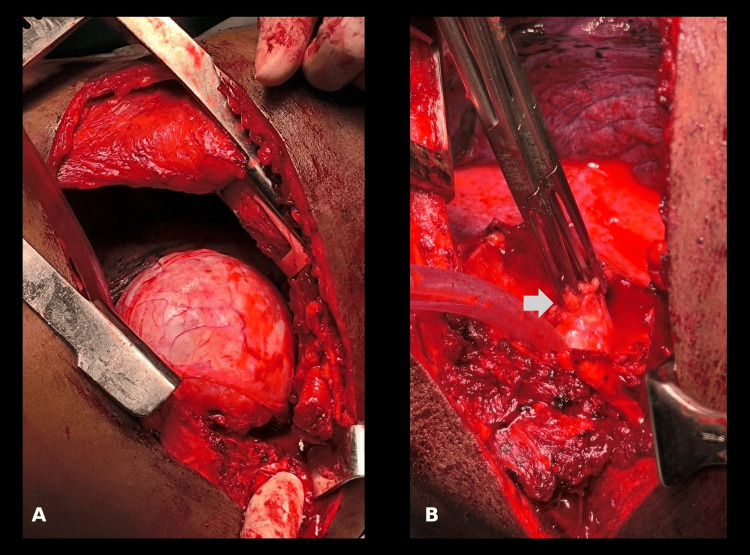
The intrathoracic portion of the dumbbell tumor visible through posterolateral thoracotomy (Panel A) and the intraspinal portion of the tumor prior to excision (Arrow - Panel B)

**Figure 3:**
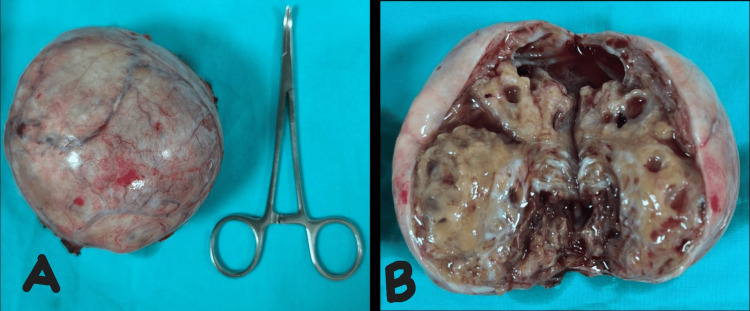
Specimen shown next to a Halsted mosquito forceps for scale. Anterior aspect of specimen (Panel A), specimen cut open showing areas of cystic degeneration (Panel B)

Following surgery, the patient had a protracted stay in the hospital due to developing a postoperative lower respiratory tract infection that needed intravenous antibiotics. At discharge, three weeks later, his neurological examination remained unchanged from his preoperative baseline. Histopathological examination revealed that, of the 80 mm x 78 mm x 50 mm intrathoracic specimen and the 35 mm x 25 mm x 20 mm intraspinal component received, a benign, low-grade, spindle cell tumor with cystic degeneration representing schwannoma was noted. The sampled station 9 lymph node was reactive. Immunohistochemistry was not available. A postoperative MRI or CT was not done since the surgery was performed in a resource-poor setting. However, a postoperative chest X-ray was performed and compared with the preoperative chest X-ray (Figure 4).

**Figure 4:**
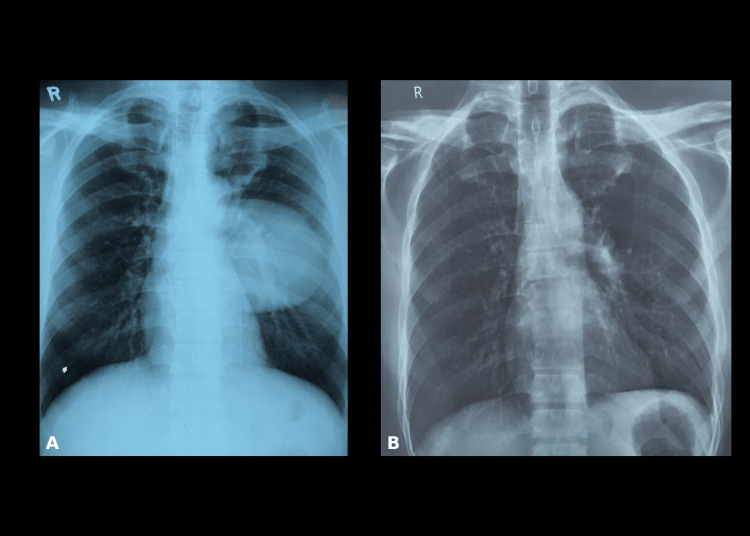
Chest x-rays of the patient Panel A - preoperative chest x-ray. Panel B - postoperative chest x-ray.

## Discussion

The differential diagnosis of a posterior mediastinal mass is led by neurogenic tumors, schwannoma, and neurofibroma. It also includes ganglioneuroma, ganglioneuroblastoma, neuroblastoma, and paraganglioma. Non-neoplastic mimics, such as neuroenteric cysts, lateral meningoceles, extramedullary hematopoiesis, and paravertebral osseous or infective lesions, should also be considered [1-3]. Schwannomas are the most common of these lesions [1,2]. They are typically slow-growing, well-encapsulated, and frequently discovered incidentally on chest imaging. When symptoms do occur, they are usually nonspecific and relate to an enlarging tumor. These symptoms include chest pain, cough, and dyspnea [1-3].

One highlight of the present case was its incidental discovery during evaluation of an unrelated respiratory illness. Despite a large thoracic dumbbell tumor and marked spinal canal narrowing, the patient had no symptoms due to mass effect, nor did he have neurological deficits. Similar thoracic dumbbell schwannomas have been reported in neurologically intact patients, including lesions with foraminal extension and cord compression detected incidentally [6,7,9]. In contrast, other reported thoracic dumbbell schwannomas, especially larger or more advanced lesions, have presented with lower limb weakness, numbness, gait disturbance, or progressive myelopathic symptoms [10,11]. With the available literature, we can infer that tumor size and the degree of spinal canal narrowing do not reliably predict the neurological status at presentation [6-11]. Thus, once a posterior mediastinal dumbbell lesion is identified, MRI to evaluate the spinal cord, even in patients who remain neurologically intact, should be considered good clinical practice [3,5].

The second highlight from this case concerns management. Gross total resection remains the treatment of choice for thoracic dumbbell tumors, as it relieves compression on neural structures and provides definitive local treatment [6,9,12]. Traditionally, these lesions have been managed through open approaches, including posterior, posterolateral, anterior, and combined thoracic-neurosurgical operations, selected according to tumor size, compartmental extension, and local expertise [6,8,12]. Although open surgery provides wide exposure, it often requires substantial paraspinal muscle dissection and bony exposure. Minimally invasive and thoracoscopic-assisted techniques have also been described in selected patients; however, experience remains limited and long-term comparative outcome data are still sparse [9,12,13].

In our patient, the intrathoracic component was approached first, followed by the intraspinal component accessed through the intervertebral foramen. The extradural intraspinal lesion was excised piecemeal to minimize traction on the spinal cord, and there was no dural tear. Given the large intrathoracic and intraspinal components, a traditional open combined approach was considered the most appropriate strategy. At the one-month postoperative review, the patient maintained his intact neurological state.

We observed a successful macroscopic surgical excision without neurological injury in our patient. This underscores the importance of a timely multidisciplinary approach. In this case, the thoracic surgical and spinal surgical teams combined to perform a complete macroscopic excision of both intrathoracic and intraspinal components in a single procedure [9,12]. This was done on an elective surgical list, and the delay to surgery was approximately 10 weeks from the initial presentation. In the meantime, preoperative optimization of the patient was done.

## Conclusions

This case highlights that thoracic dumbbell schwannomas may be discovered incidentally and can remain neurologically silent despite substantial intraspinal extension and significant spinal canal narrowing. The absence of focal neurological deficits should not lessen concern when imaging demonstrates a posterior mediastinal dumbbell lesion. Early MRI, timely referral, and coordinated multidisciplinary planning are essential to define the full extent of the disease and to guide safe resection. In our patient, individualized surgical management achieved complete macroscopic excision while preserving an intact neurological status.
